# Accuracy of continuous glucose monitoring during noncardiac surgery: a prospective, blinded observational multicentre cohort study

**DOI:** 10.1016/j.bja.2025.05.057

**Published:** 2025-07-24

**Authors:** Henrike Janssen, Priyanthi Dias, Sanjali Ahuja, Saja Alharbi, Louise Hiller, Kamran Khan, Karthik Iyer, Ashok Sundar, Islam Abousharkh, Caroline Thomas, Shaman Jhanji, Nick S. Oliver, Gareth L. Ackland, GlucoVITAL Investigators

**Affiliations:** 1 Translational Medicine and Therapeutics, William Harvey Research Institute, Queen Mary University of London, London, UK; 2 King Saud bin Abdulaziz University for Health Sciences, Riyadh, Saudi Arabia; 3 King Abdullah International Medical Research Center, Riyadh, Saudi Arabia; 4 Warwick Clinical Trials Unit, University of Warwick, Warwick, UK; 5 University Hospitals Birmingham NHS Trust, Birmingham, UK; 6 Croydon University Hospital, Croydon, Croydon, UK; 7 St James's University Hospital, Leeds, UK; 8 Department of Anaesthesia, Perioperative Medicine and Critical Care, Royal Marsden Hospital, London, UK; 9 Department of Metabolism, Digestion and Reproduction, Faculty of Medicine, Imperial College London, London, UK

**Keywords:** complications, glucose monitoring, hyperglycaemia, noncardiac surgery, perioperative care

## Abstract

**Background:**

Hyperglycaemia after noncardiac surgery is rarely detected outside of the critical care environment, yet occurs commonly and is associated with excess complications including infections and myocardial injury. Systematic, prospectively collected data regarding the accuracy of continuous glucose monitoring commenced immediately before surgery and throughout the early perioperative period are lacking.

**Methods:**

We prospectively enrolled patients aged >50 yr undergoing noncardiac surgery who required at least 24 h of hospital stay. We used real-time continuous glucose monitoring (Dexcom G7 sensor, placed in the upper outer arm) (Dexcom, San Diego, CA, USA) with reference values from arterial blood glucose measurements by amperometry. The primary outcome was the overall mean difference (bias) before surgery, at end of surgery, and 24 h after surgery (Bland–Altman analysis). Secondary outcomes included the mean absolute relative difference and surveillance error grid analyses.

**Results:**

We compared paired blood (73% arterial) and continuous glucose monitoring glucose values at each prespecified timepoint in 118 participants (64/118 [54%] female; mean age: 66 [range: 51–89] yr; 25% with diabetes mellitus). The overall bias between continuous glucose monitoring and blood glucose from measurements at each of the three timepoints in the first 24 h after induction of anaesthesia was 0.38 mM (95% confidence interval [95% CI]: 0.23–0.53; *n*=340 paired readings). Bias decreased from before the start of surgery (1.08 mM [95% CI: 0.87–1.29]; *n*=116) to 0.15 mM at the end of surgery (95% CI: −0.15 to 0.46; *n*=113). Mean absolute relative difference ranged from 12.0% to 18.3%. Error grid analyses found that >98% continuous glucose monitoring values were within acceptable risk ranges.

**Conclusions:**

The accuracy of state-of-the-art continuous glucose monitoring is sufficient for perioperative use and could enhance perioperative surveillance of dysglycaemia.

**Clinical trial registration:**

ISRCTN46862025.

**Editor's key points**

• Hyperglycaemia is common after noncardiac surgery and is associated with increased complications including infection and myocardial injury.
• The accuracy of a continuous glucose monitoring device placed immediately before surgery and measured throughout the early perioperative period was compared with arterial blood glucose measurements in 118 participants with or without diabetes mellitus.
• The accuracy of continuous glucose monitoring was within acceptable limits of the gold-standard, blood glucose measurements.
• Continuous glucose monitoring values accurately measure glucose concentration which could help clarify the role of perioperative hyperglycaemia in developing organ injury after noncardiac surgery.

Stress hyperglycaemia is a prototypical response to tissue injury including surgery^1^ and is associated with an increased risk of postoperative complications.^2^ Before surgery, even modest elevations in fasting plasma glucose (>6.4 mM) are associated with a higher risk of developing postoperative cardiovascular outcomes.^3^ However, there is almost double the risk of serious complications in non-diabetic individuals than in patients with established diabetes mellitus who have the same level of perioperative hyperglycaemia sustained.^4^ Given the extent of insulin resistance in the general population,^5^ non-diabetic patients undergoing major surgery likely experience injurious hyperglycaemic periods that remain undetected.^4^ In surgical patients, insulin resistance is common as a consequence of a large number of patients undergoing surgery for the resection of malignant tumours.^6^ Insulin resistance in cancer patients is characterised by increased hepatic glucose production and gluconeogenesis, and unlike type 2 diabetes mellitus, normal fasting glucose with high, normal, or low levels of insulin.^7^ Perioperative exposure to transient hyperglycaemia fuels systemic inflammation,^8^ endothelial dysfunction,^9^ and acute leucocyte dysfunction,^10^ all of which are pivotal mechanisms for causing organ injury after major surgery.

Until interstitial glucose monitoring became widely available, detection of perioperative dysglycaemia at scale in noncardiac surgical patients outside a critical care environment has been challenging. With the advent of continuous glucose monitoring, two studies have reported intraoperative continuous glucose monitoring in ∼20 surgical patients without diabetes mellitus^11^ and 20 patients with pre-diabetes mellitus.^12^ The latter study commenced continuous glucose monitoring 10 days before surgery,^12^ which is logistically challenging for many perioperative settings. However, the accuracy and reliability of continuous glucose monitoring when sensors are sited at the time of induction of anaesthesia have not been undertaken, which is critical to advance both mechanistic insights and the pragmatic real-world use of continuous glucose monitoring in the perioperative period.^13^ This is also required to resolve longstanding concerns regarding the impact of vasopressor use and electrocautery on continuous glucose monitoring reliability and accuracy.

As part of the ongoing mechanistic study GlucoVITAL (ISRCTN: 46862025), we prospectively compared continuous glucose monitoring measurements with gold-standard blood glucose values systematically before and at the end of surgery and 24 h after surgery to establish the reliability and accuracy of the latest generation continuous glucose monitoring system.

## Methods

This calibration study was conducted in accord with Standards for Reporting of Diagnostic Accuracy Studies (STARD) 2015 guidelines.^14^ The 118 participants included in this analysis are a subset of the ongoing GlucoVITAL trial (ISRCTN: 46862025). Six centres contributed to this planned calibration substudy, for which a statistical analysis plan was published on October 8, 2024 (www.qmul.ac.uk/ccpmg/sops--saps/statistical-analysis-plans-saps; Supplementary material). The study was approved by the London-Fulham Research Ethics Committee (23/PR/0677). An independent Trial Monitoring Committee and steering committee oversee the GlucoVITAL trial. Potential participants were identified by their surgical or perioperative teams. Inclusion criteria for the GlucoVITAL trial were age ≥50 yr, elective major noncardiac surgery under general anaesthesia, and written informed consent for trial participation. Exclusion criteria for the GlucoVITAL trial were known contraindication to either total i.v. anaesthesia (TIVA) or inhalation anaesthesia, clinician refusal, participant not expected to survive for 30 days, and previous participation in VITAL trial.

### Blood glucose measurements

To compare continuous glucose monitoring values with blood glucose values, we used hospital-specific blood gas analysers (Supplementary Table S1), because in critically ill adult patients, the accuracy of blood glucose measurements with arterial blood gas analysers is higher than that of measurements with glucose meters using capillary blood.^15^ Venous and capillary samples were used if arterial samples were not clinically appropriate as the bias between arterial and capillary values is low in the perioperative phase.^16^

### Continuous glucose monitoring

The Dexcom G7 sensor patch^17^ was placed on the upper outer arm immediately after induction of anaesthesia by trained research staff. After the 20-min warm-up period, Dexcom receiver activity was confirmed by research staff. Receivers were secured to the bed of each patient in an opaque case to ensure blinding to clinical staff throughout the perioperative period. Sensor data were recorded for the duration of the hospital stay, or until the 10 days expiry period of each sensor. Receiver alarms were deactivated.

### Perioperative management

Aside from the randomised assignment to maintenance of anaesthesia in line with the VITAL trial,^18^ clinical care was left to the discretion of attending teams, including fluid therapy and analgesic regimens. Intraoperative temperature management was in accord with UK National Institute for Clinical Excellence guidelines to prevent perioperative hypothermia.^19^

### Primary outcome

The primary outcome was the mean difference between paired continuous glucose monitoring and blood glucose values obtained before surgery, at the end of surgery, and 24 h after surgery.

### Secondary outcomes

Secondary outcomes included the mean absolute relative difference (MARD) and surveillance error grids constructed using the latest Diabetes Technology Society Error Grid,^20^ plus surveillance, Parkes, and Clarke error grids to assess clinical accuracy and illustrate the clinical consequences of sensor/reference deviation.^21^

### Safety measures

We recorded sensor-specific adverse effects (local skin irritation, bruising, bleeding), alongside the number of patients in whom sensor measurements were made as a metric of usability.

### Statistical analysis

Participants with missing paired outcome data were excluded from the analysis. For the analysis of the primary outcome, each secondary outcome, and all process measures, summary statistics (*n* [%]) for count data, point estimates, and measure of variation for continuous data) are presented. For the primary outcome, we calculated bias (mM), with lower (bias−1.96∗SDRD) and upper (bias+1.96∗SDRD) 95% limits of agreement, where standard deviation of relative difference (SDRD) was calculated from each continuous glucose monitoring value and blood glucose reference value. We calculated MARD by averaging the absolute values of relative differences between continuous glucose monitoring and blood glucose monitoring system measurement results and corresponding comparison method results.^22^ Full details of the Diabetes Technology Society error grid calculations are available online.^20^ We also conducted a full sensitivity analysis for surgical patients with diabetes mellitus alone. As an exploratory analysis, we also assessed the primary outcome in patients who received vasopressor infusions during and after surgery in the first 24 h.

A two-sided *P*-value was used for all analyses, with a significance level set at 5%. All analyses were conducted using R version 4.3.2 (R Foundation for Statistical Computing, Vienna, Austria),^23^ NCSS 2023 (NCSS, LLC, Kaysville, UT, USA; ncss.com/software/ncss), or both.

### Sample size estimation

We calculated that this substudy would require a minimum sample size of 110 paired samples to achieve a power of 90% and a level of significance of 5% (two-sided) in order to detect a mean of the differences of 1 mM between pairs, assuming the standard deviation of the differences to be 0.8 mM with a clinically acceptable difference of 3 mM (limit of agreement; R version 4.3.2).

## Results

### Participant characteristics

**Fig 1:**
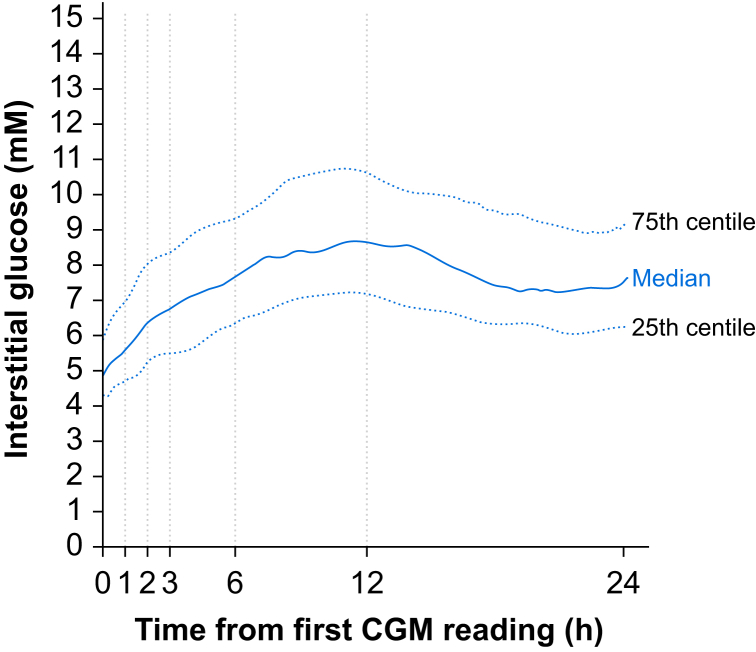
Changes in interstitial glucose measurements in patients when sensors (Dexcom G7) were placed before surgery. Median (25th–75th percentile) values for interstitial glucose every 5 min for the first 24 h after sensor placement. CGM, continuous glucose monitoring.

This planned substudy was conducted from September 28, 2023, to September 25, 2024, using data from 123 consecutively enrolled patients from six trial sites (Supplementary Fig. S1). The majority of surgery was undertaken for intraabdominal cancer (Table 1) in older (mean [sd] age: 66 [9] yr), higher risk patients, as reflected by ∼50% assessed as ASA physical status ≥3. By the morning after surgery, five sensors were either not providing any or reliable data or were not applied to the patient (*n*=2) because of a trial deviation in the parent trial VITAL (Supplementary Fig. S1). Seven further patients did not have a complete set of blood glucose–continuous glucose monitoring paired readings at each timepoint, leaving 111 participants with complete 24-h data. Arterial blood was the source of blood glucose measurements in 73.7% of samples. Over the course of the first 24 h, interstitial glucose values increased (Fig. 1), with 447/31602 readings (1.4%) registered below 3.1 mM, the low limit alarm set by the Dexcom G7 sensor system.

**Table 1:** Patient and surgery characteristics. Data are presented as *n* (%) unless otherwise indicated. ACEi, angiotensin converting enzyme inhibitor; ARB, angiotensin-II receptor blocker; GI, gastrointestinal; DPP4, dipeptidyl peptidase-4; GLP-1, glucagon-like peptide-1; SGLT2i, sodium-glucose cotransporter-2 inhibitor.

Age (yr), mean (sd)	66 (9)
Female sex	53 (45)
ASA physical status ≥3	57 (49)
Frailty (vulnerable or frail)	7 (6)
Body mass index	
≥30 kg m^−2^	34 (29)
≤18.5 kg m^−2^	3 (3)
Patients with diabetes mellitus	29 (25)
Anti-diabetic medications	
Insulin	6 (5)
Metformin	21 (18)
DPP4	6 (5)
GLP-1 or SGLT2i	6 (5)
Sulfonylurea	5 (4)
Cardiovascular medications	
ACEi	21 (18)
ARB	15 (13)
Beta-blocker	14 (12)
Calcium channel blocker	22 (19)
Diuretic	11 (9)
Statin	42 (36)
Anticoagulants	20 (17)
Steroids	12 (10)
Immunomodulatory agents	3 (3)
Surgery for cancer	98 (84)
Type of surgery	
Colorectal	28 (24)
Gynae-oncologic	25 (21)
Hepatobiliary	17 (15)
Upper GI	22 (19)
Duration of surgery, min, mean (sd)	351 (164)
Dexamethasone use	84 (68)
Insulin use	18 (15)

### Safety of continuous glucose monitoring

There were no sensor safety issues recorded, with no local skin irritation, bleeding, infections, or bruising/hematomas noted. Both monopolar and bipolar electrocautery was used during surgery at the discretion of the surgical team. We could not establish any time relationship between use of electrocautery and sensor disruption.

### Primary outcome

**Fig 2:**
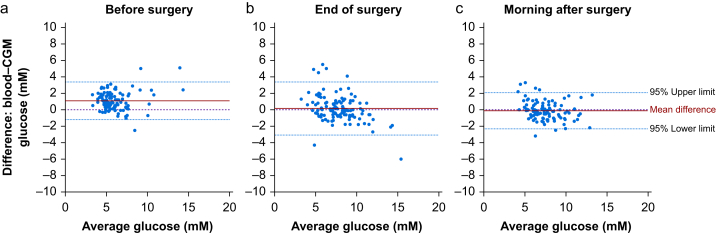
Primary outcome: bias and accuracy for perioperative CGM. Modified Bland–Altman plot of paired glucose values from CGM and reference blood glucose values (a) before surgery, (b) at the end of surgery, and (c) on the morning after surgery. Mean difference is indicated by the solid red line in each panel. Lower (bias−1.96∗SDRD) and upper 95% limits of agreement (bias+1.96∗SDRD), where SDRD (standard deviation of relative difference) was calculated from each CGM value and blood glucose reference value. CGM, continuous glucose monitoring.

The overall bias between continuous glucose monitoring and blood glucose measurements made within the first 24 h after induction of anaesthesia was 0.38 mM (95% confidence interval [95% CI]: 0.23–0.53; *n*=340 paired readings). When continuous glucose monitoring sensors were first placed before the start of surgery, bias was 1.08 mM (95% CI: 0.87–1.29; *n*=116 paired readings); that is, continuous glucose monitoring readings were lower compared with blood glucose values before surgery (Fig. 2a). By the end of surgery, bias reduced to 0.15 mM (95% CI: −0.15 to 0.46; *n*=113 paired readings; Fig. 2b). On the morning after surgery, ∼24 h after sensor placement (Fig. 2c), bias was −0.11 mM (95% CI: −0.33 to 0.10; *n*=111 paired readings).

### Secondary outcomes

**Fig 3:**
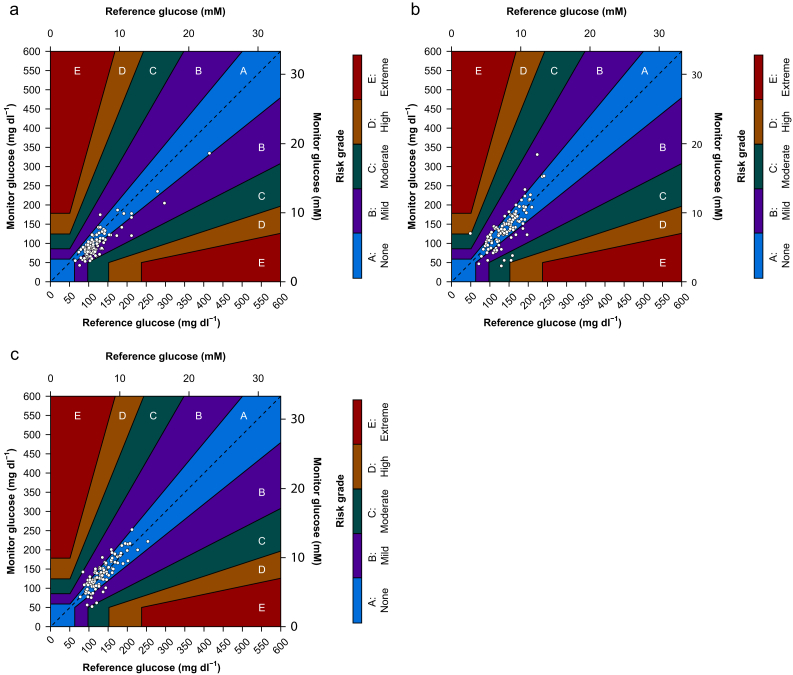
Secondary outcomes: Diabetes Technology Society (DTS) error grid analysis. DTS error grid for paired continuous glucose monitoring and reference blood glucose values (a) before surgery, (b) at the end of surgery, and (c) on the morning after surgery. Different colours reflect degree of risk as quantified in the vertical heatmap.

MARD decreased from 18% when the sensors were placed before surgery to 12% the morning after surgery (Table 2). The Diabetes Technology error grid analyses at each prespecified timepoint (Fig. 3) found that >98% values were within low–moderate risk ranges for each timepoint at which paired continuous glucose monitoring–blood glucose values were recorded (Table 3). Additional error grid analyses, including the Parkes and Clarke grids, similarly showed that >99.2% of continuous glucose monitoring values were within acceptable risk ranges (Supplementary Figs. S5–S8), with <1% deemed greater than errors of moderate risk (Table 3). Additional secondary analyses of 15/15 values are provided in Supplementary Table S2.

**Table 2:** Bias and accuracy overall and at each prespecified timepoint. LoA is calculated as lower (bias−1.96∗SDRD) and upper (bias+1.96∗SDRD) 95% LoA. SDRD is calculated from each continuous glucose monitoring value and blood glucose reference value. LoA, limits of agreement; MARD, mean absolute relative difference; SDRD, standard deviation of relative difference.

	Absolute bias (sd), mM	Relative bias, %	Lower 95% LoA, %	Upper 95% LoA, %	MARD, %	SDRD, %
Overall	0.38 (0.23–0.53)	−5.4	−46.0	35.3	15.3	20.7
Before surgery	1.08 (0.87–1.29)	−16.0	−46.2	14.3	18.3	15.3
End of surgery	0.15 (−0.15 to 0.46)	−1.5	−50.3	47.4	15.6	24.6
Morning after surgery	−0.11 (−0.33 to 0.10)	1.7	−30.9	34.3	12.0	16.5

**Table 3:** Error grid comparisons. Data are presented as *n* (%), for each timepoint before, after, and 24 h after surgery. Clarke, Clarke error grid; DTS, Diabetes Technology Society error grid; Parkes, Parkes error grid; SEG, surveillance error grid.

	DTS	SEG	Parkes	Clarke
Before surgery				
A: No risk	69 (59.5)	72 (60.1)	74 (63.8)	69 (59.4)
B: Mild risk	47 (40.5)	44 (37.9)	42 (36.2)	46 (39.7)
C: Moderate risk	0	0	0	0
D: High risk	0	0	0	0
E: Extreme risk	0	0	0	0
End of surgery				
A: No risk	85 (75.2)	91 (80.5)	90(79.6)	85 (75.2)
B: Mild risk	23(20.4)	17 (15.0)	22 (19.5)	27 (23.9)
C: Moderate risk	4 (3.5)	5 (4.4)	0	0
D: High risk	1 (0.9)	0	1 (0.9)	1 (0.9)
E: Extreme risk	0	0	0	0
Morning after surgery				
A: No risk	92 (82.9)	96 (60.2)	99 (89.2)	92 (82.9)
B: Mild risk	17 (15.3)	14 (12.6)	12 (10.8)	19 (17.1)
C: Moderate risk	2 (1.8)	1 (0.9)	0	0
D: High risk	0	0	0	0
E: Extreme risk	0	0	0	0

### Sensitivity analyses

For the 29 individuals treated for diabetes mellitus (type 2) before surgery, mean blood glucose values in the first 24 h after continuous glucose monitoring sensor placement were 8.9 mM (95% CI: 8.3–9.5). For patients with diabetes mellitus, the overall bias between continuous glucose monitoring and blood glucose for the three paired measurements made within the first 24 h after induction of anaesthesia was 0.51 mM (95% CI: 0.13–0.90; Supplementary Fig. S2).

For 95/116 patients who received vasopressor infusions during or after surgery in the first 24 h, bias (0.36 mM [95% CI: 0.20–0.53]) and accuracy were similar to those who did not require haemodynamic support (Supplementary Fig. S3). Paired data analyses including beyond the 24-h period showed a bias of 0.07 mM (95% CI: −0.03 to 0.16; Supplementary Fig. S4).

## Discussion

Immediate placement of the latest generation continuous glucose monitoring sensor in patients undergoing major noncardiac surgery was safe and provided data of sufficient accuracy for perioperative use. The early higher bias rapidly resolved by the end of surgery, indicating that the short warm-up period of this generation of sensors (Dexcom G7) is unaffected by many well-documented potential perioperative confounders, including relative hypothermia, extracellular fluid accumulation, reduced blood supply to upper arm muscle and other tissues, dysoxia, and changes in glucose as part of the stress response. As the purpose of this study was to establish the accuracy of continuous glucose monitoring data, our data provide the key first step in progressing to systematically re-examine at scale the role that stress hyperglycaemia plays in fuelling organ dysfunction after tissue trauma.

Compared with a previous study of 20 patients with pre-diabetes mellitus where continuous glucose monitors were placed several days before surgery,^12^ our approach of siting continuous glucose monitoring sensors was necessary to facilitate the main parent trial. Moreover, in the current surgical environment across the UK, and we suspect many other healthcare settings, placement of continuous glucose monitoring monitors so many days before surgery is not feasible to operationalise and is therefore not generalisable. If continuous glucose monitoring becomes used more widely in perioperative medicine, establishing its accuracy in as close to real-world conditions as practical is a key first step. The US Food and Drug Administration (FDA) approved the use of continuous glucose monitoring systems for treatment of patients in hospital settings and other healthcare facilities during COVID-19 and thereafter.

Currently, the Dexcom G7 is contraindicated in patients undergoing MRI, with caution over treatment involving diathermy. Our data add to this real-world knowledge by indicating that sensor failure is unlikely in surgical scenarios included in the GlucoVITAL trial when diathermy is used. Both monopolar and bipolar electrocautery was used during surgery at discretion of the surgical team. The continuous glucose monitoring sensor was always placed on the upper left or right arm. We could not establish a time relationship between use of electrocautery and sensor failure. However, surgical procedures were predominately below the thorax which could have influenced the low failure rate in sensors observed.

In contrast to previous reports in patients admitted to ICU after surgery,^24^ we did not record detachment of sensors or disturbances due to arm positioning. The research staff took great care to position the sensors in a way unimpeded by arm wrapping or extension. It seems that additional sites are sufficient to provide accurate continuous glucose monitoring data, including the infraclavicular region^25^ which was used in 61 patients requiring intensive care after major abdominal surgery including solid organ transplantation.^24^ Similarly, we did not observe continuous glucose monitoring recording instability attributable to the use of (bipolar) electrocautery. In contrast to a previous (unpublished) pilot project that examined continuous glucose monitoring before and during surgery,^24^ we did not find interference with other Bluetooth devices or other equipment including patient warming systems. This might reflect the use of the latest generation Dexcom G7 sensor, in contrast to reports using the earlier G6 sensor system.

In contrast to metrics used for management of diabetes mellitus, the key issue in our study was to determine whether continuous glucose monitoring provides sufficient accuracy from the start of the perioperative period. Although we could have calibrated each sensor, the range of bias found would not alter clinical behaviour by anaesthesiologists. Using all available Surveillance Error Grid analyses, our data show that >99.2% perioperative continuous glucose monitoring values were within acceptable risk ranges, with <1% deemed greater than errors of mild risk. These findings are in accord with the accuracy levels reported by the manufacturer for conditions of usual use.

We acknowledge that the MARD values we found were higher than the value provided by Dexcom in normal use. However, in this acute first 24 h of sensor use,^26^ it is debatable whether MARD is a clinically relevant metric for perioperative care.^27^ MARD is typically high during the initial phase after sensor insertion, and declines thereafter. Thus, MARD metrics critically depend on the phase from which data are used for MARD calculation. MARD calculations for all usage days would provide more instructive information about the changes in analytical performance of different continuous glucose monitoring systems, but are usually reduced to a single report over the whole period of sensor use.^27^

Our preplanned sensitivity analyses failed to find appreciable difference in bias for patients with diabetes mellitus or receiving vasopressor infusions for haemodynamic control (both during and after surgery) compared with respective controls. These data are relevant for ICU populations in whom acute critical illness frequently requires the early use of vasoactive drugs. This has limited the certainty with which continuous glucose monitoring can be used in this setting. Our prospectively collected data initiated before the introduction of vasopressors suggests that this concern is likely not impactful. However, we do acknowledge that the doses of vasopressor infused in our study, although in the range of ICU use, were often lower than those used in the most seriously critically ill patients.

Continuous glucose monitoring might facilitate mechanistic understanding into perioperative hyperglycaemia by accurately capturing glucose variability from early in the surgical episode.^13^ These data represent a key step in systematically re-examining the role that stress hyperglycaemia might play in fuelling organ dysfunction after tissue trauma. Large observational cohort series using blood glucose monitoring have identified that substantial numbers of patients without an established diagnosis of diabetes mellitus experience undetected hyperglycaemia.^4^ Establishing continuous glucose monitoring as being sufficiently accurate will now enable quantification of glucose changes in individuals without diabetes mellitus, who have twice the number of serious complications for the same level of hyperglycaemia sustained by patients with established diabetes mellitus.^4^ The targeted use of continuous glucose monitoring in patients that the GlucoVISION study identified before surgery as having substantially higher risk of developing postoperative cardiovascular outcomes might provide additional mechanistic insights,^3^ as we are undertaking in the GlucoVITAL study. These calibration data confirm that it should be possible to determine at scale glucose levels in the large number of patients at risk of hyperglycaemia undergoing major surgery, and therefore map glycaemic profiles to clinical outcomes as a new indication for continuous glucose monitoring.^28^

A major strength of this multicentre study is that all measurements were blinded, with clinical staff and patients remaining unaware of continuous glucose monitoring data. The multicentre design enrolling a range of noncardiac surgical patients suggests the data are likely to be generalisable. Systematic collection of paired data at predefined time points minimised variability. Use of both the latest generation continuous glucose monitoring system and the Diabetes Technology Society error grid analysis provides state-of-the-art information.^20^ We elected not to calibrate continuous glucose monitoring sensors with blood glucose values, as this would be a major deviation in usual clinical care. We cannot rule out that calibration early on might have limited the bias observed for the first datapoint (before surgery). However, given that bias reduced rapidly by the end of surgery, this appears unlikely to have a meaningful clinical impact moving forward.

In summary, perioperative accuracy of state-of-the-art continuous glucose monitoring is sufficient for clinical use. These data suggest that continuous glucose monitoring values might facilitate mechanistic understanding of the role of perioperative hyperglycaemia in developing organ injury after noncardiac surgery.

## Authors’ contributions

Collected the data and wrote the first draft of the manuscript: HJ, LH, AS, KI, IA, PD, CT, SJ, GLA

Contributed to discussion: HJ, LH, AS, KI, IA, PD, CT, SJ, GLA, NSO

Reviewed and edited the manuscript: NSO

Provided statistical support and design: LH, KK

Approved the final version of the manuscript: all authors

## Funding

UK National Institute for Healthcare Research (Efficacy and Mechanism Evaluation grant NIHR154842); British Heart Foundation (programme grant RG/19/5/34463 to GLA); National Institute for Healthcare Research Advanced Fellowship (NIHR300097 to GLA); British Journal of Anaesthesia/Royal College of Anaesthetists (UK Centenary grant 2024-27 to HJ); National Institute for Healthcare Research Biomedical Research Centre at The Royal Marsden NHS Trust and Institute of Cancer Research, London (SJ); Dexcom, Medtronic, and Roche Diabetes (to NSO).

## Declarations of interest

GLA is an editor of the *British Journal of Anaesthesia*. NSO has participated in advisory groups for Dexcom, Medtronic, and Roche Diabetes, and has received fees for speaking from Sanofi, Dexcom, Tandem, Medtronic, and Roche Diabetes. The other authors declare that they have no conflicts of interest.
