# Supplementary material for: Accuracy of continuous glucose monitoring during noncardiac surgery: a prospective, blinded observational multicentre cohort study
Source: Br J Anaesth. 2025 Jul 24;135(4):912–9. doi: 10.1016/j.bja.2025.05.057 (PMC12674053; doi:10.1016/j.bja.2025.05.057)

**Accuracy of Continuous Glucose Monitoring commenced immediately before noncardiac surgery: prospective, blinded observational multicentre cohort study.**

glucoVITAL Investigators -Supplementary data files

Contents

[STARD statement. 2](#_Toc195709994)

[Sample size calculation 3](#_Toc195709995)

[Supplementary Table 1. Apparatus used for blood glucose checks in each center. 4](#_Toc195709996)

[Supplementary Table 2. %15/15 values. 5](#_Toc195709997)

[Supplementary figure 1. Study flow diagram. 6](#_Toc195709998)

[Supplementary Figure 2. Sensitivity analysis for primary outcome in patients with diabetes mellitus. 7](#_Toc195709999)

[Supplementary Figure 3. Sensitivity analysis for primary outcome in patients who received vasopressor infusions during the first 24h after sensor placement. 7](#_Toc195710000)

[Supplementary Figure 4. Paired comparisons with CGM for 873 blood glucose measurements including and beyond 24h. 8](#_Toc195710001)

[Supplementary Figure 5. Comprehensive error grid analyses for pre-surgery paired comparisons. 9](#_Toc195710002)

[Supplementary Figure 6. Comprehensive error grid analyses for end of surgery paired comparisons. 10](#_Toc195710003)

[Supplementary Figure 7. Comprehensive error grid analyses for morning after surgery paired comparisons. 11](#_Toc195710004)

[Supplementary Figure 8. DTS error grid analyses for all paired comparisons throughout surgical period. 12](#_Toc195710005)

# STARD statement.

|  | **Section & Topic** | **No** | **Item** | **Reported on page #** |
| --- | --- | --- | --- | --- |
|  |  |  |  |  |
|  | **TITLE OR ABSTRACT** |  |  |  |
|  |  | **1** | Identification as a study of diagnostic accuracy using at least one measure of accuracy  (such as sensitivity, specificity, predictive values, or AUC) | 3 |
|  | **ABSTRACT** |  |  |  |
|  |  | **2** | Structured summary of study design, methods, results, and conclusions  (for specific guidance, see STARD for Abstracts) | 3 |
|  | **INTRODUCTION** |  |  |  |
|  |  | **3** | Scientific and clinical background, including the intended use and clinical role of the index test | 5-6 |
|  |  | **4** | Study objectives and hypotheses | 6 |
|  | **METHODS** |  |  |  |
|  | *Study design* | **5** | Whether data collection was planned before the index test and reference standard were performed (prospective study) or after (retrospective study) | n/a |
|  | *Participants* | **6** | Eligibility criteria | 7 |
|  |  | **7** | On what basis potentially eligible participants were identified  (such as symptoms, results from previous tests, inclusion in registry) | 7 |
|  |  | **8** | Where and when potentially eligible participants were identified (setting, location and dates) | 7 |
|  |  | **9** | Whether participants formed a consecutive, random or convenience series | 7 |
|  | *Test methods* | **10a** | Index test, in sufficient detail to allow replication | 8 |
|  |  | **10b** | Reference standard, in sufficient detail to allow replication | 8 |
|  |  | **11** | Rationale for choosing the reference standard (if alternatives exist) | 8 |
|  |  | **12a** | Definition of and rationale for test positivity cut-offs or result categories  of the index test, distinguishing pre-specified from exploratory | n/a |
|  |  | **12b** | Definition of and rationale for test positivity cut-offs or result categories  of the reference standard, distinguishing pre-specified from exploratory | n/a |
|  |  | **13a** | Whether clinical information and reference standard results were available  to the performers/readers of the index test | 8 |
|  |  | **13b** | Whether clinical information and index test results were available  to the assessors of the reference standard | 8 |
|  | *Analysis* | **14** | Methods for estimating or comparing measures of diagnostic accuracy | 9 |
|  |  | **15** | How indeterminate index test or reference standard results were handled | 9 |
|  |  | **16** | How missing data on the index test and reference standard were handled | 9 |
|  |  | **17** | Any analyses of variability in diagnostic accuracy, distinguishing pre-specified from exploratory |  |
|  |  | **18** | Intended sample size and how it was determined | 10 |
|  | **RESULTS** |  |  |  |
|  | *Participants* | **19** | Flow of participants, using a diagram | suppl |
|  |  | **20** | Baseline demographic and clinical characteristics of participants | Table 1 |
|  |  | **21a** | Distribution of severity of disease in those with the target condition | n/a |
|  |  | **21b** | Distribution of alternative diagnoses in those without the target condition | n/a |
|  |  | **22** | Time interval and any clinical interventions between index test and reference standard | 11 |
|  | *Test results* | **23** | Cross tabulation of the index test results (or their distribution)  by the results of the reference standard | 11-12 |
|  |  | **24** | Estimates of diagnostic accuracy and their precision (such as 95% confidence intervals) | 11-12 |
|  |  | **25** | Any adverse events from performing the index test or the reference standard | 11 |
|  | **DISCUSSION** |  |  |  |
|  |  | **26** | Study limitations, including sources of potential bias, statistical uncertainty, and generalisability | 15 |
|  |  | **27** | Implications for practice, including the intended use and clinical role of the index test | 15 |
|  | **OTHER INFORMATION** |  |  |  |
|  |  | **28** | Registration number and name of registry | 7 |
|  |  | **29** | Where the full study protocol can be accessed | 7 |
|  |  | **30** | Sources of funding and other support; role of funders | 16 |
|  |  |  |  |  |

# Sample size calculation

The study would require a minimum sample size of 110 paired samples to achieve a power of 90% and a level of significance of 5% (two sided), for detecting a mean of the differences of 1 mmol.l^-1^ between pairs, assuming the standard deviation of the differences to be 0.8 mmol.l^-1^ and a clinically acceptable difference of 3mmol (limit of agreement).


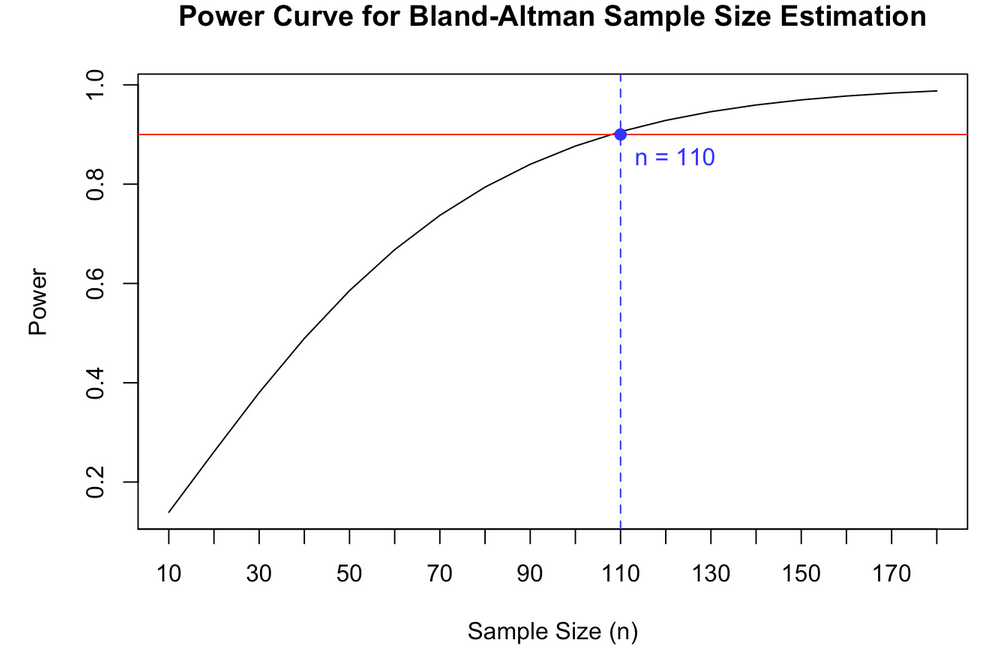


# Supplementary Table 1. Apparatus used for blood glucose checks in each center.

| **Hospital** | **Blood gas analyzer** |
| --- | --- |
| Barts Health/Royal London Hospital | ABL90 Flex (Radiometer) |
| Royal Marsden | ABL90 Flex (Radiometer) |
| University Hospitals Birmingham | Roche COBAS^®^ b 221 <6> (Roche Diagnostics) |
| Croydon University Hospital | ABL90 Flex Plus (Radiometer) |
| St James's University Hospital, Leeds | RAPIDPoint® 500e Blood Gas System (Siemens Healthineers) |

# Supplementary Table 2. %15/15 values.

For analytical accuracy, the FDA (Over-the-Counter BGM) requires ≥ 95% of monitor measurements within ±15% of reference.

|  | **Before surgery** | | **End of surgery** | | **Morning after surgery** | |
| --- | --- | --- | --- | --- | --- | --- |
| **Range** | n | % | n | % | n | % |
| **<= 5 %** | 15 | 12.70% | 25 | 21.60% | 31 | 27.70% |
| **> 5 - 10 %** | 22 | 18.60% | 27 | 23.30% | 24 | 21.40% |
| **> 10 - 15 %** | 17 | 14.40% | 26 | 22.40% | 23 | 20.50% |
| **> 15 - 20 %** | 15 | 12.70% | 11 | 9.50% | 15 | 13.40% |
| **> 20 - 25 %** | 13 | 11.00% | 9 | 7.80% | 9 | 8.00% |
| **> 25 - 30 %** | 9 | 7.60% | 6 | 5.20% | 4 | 3.60% |
| **> 35 - 40 %** | 11 | 9.30% | 4 | 3.40% | 2 | 1.80% |
| **> 40 %** | 16 | 13.60% | 8 | 6.90% | 4 | 3.60% |

# Supplementary figure 1. Study flow diagram.

All CGM numbers are referenced to original n=123.

For blood glucose samples, sequential denominator is used for number of samples obtained at each pre-specified timepoint.


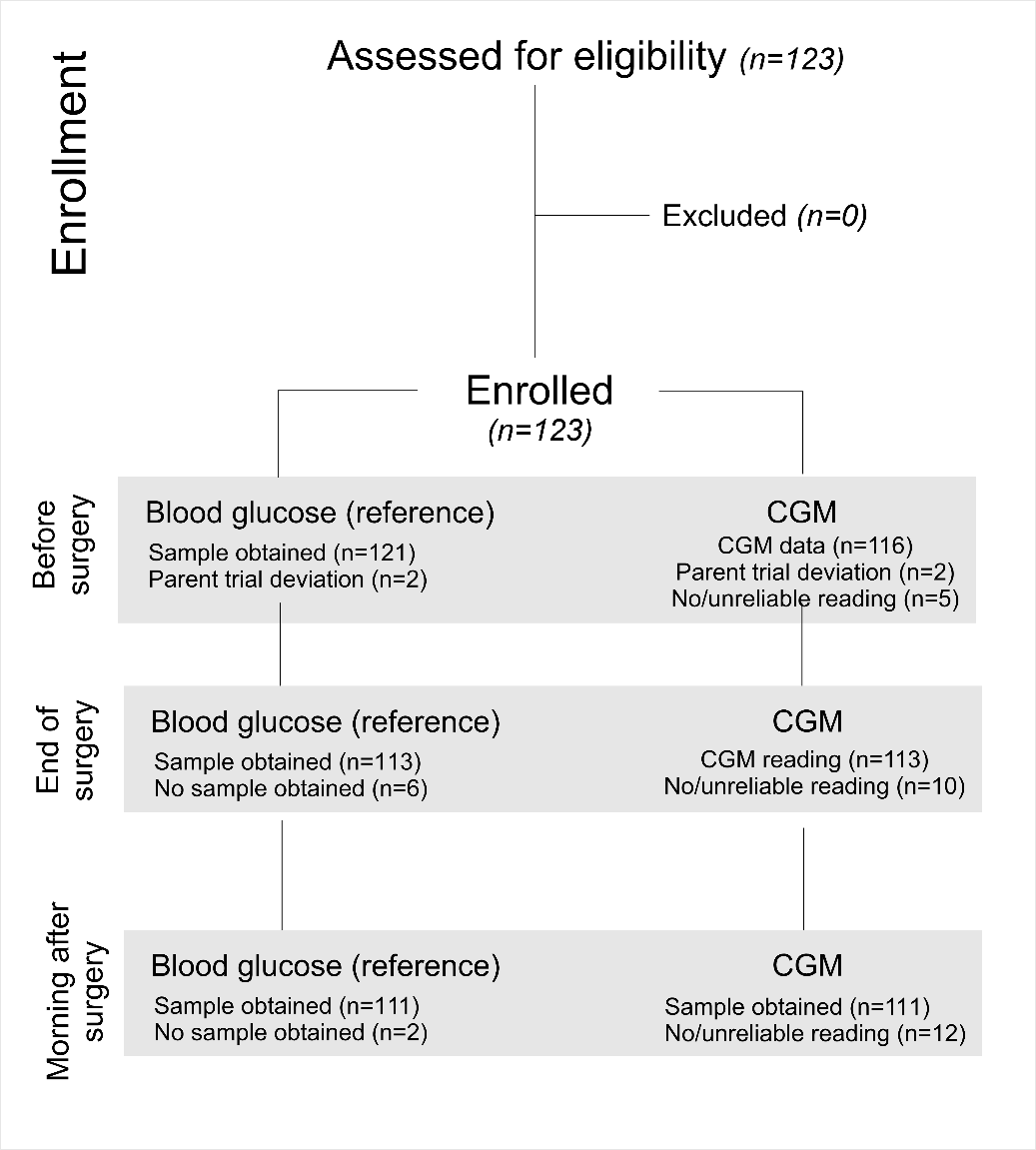


# Supplementary Figure 2. Sensitivity analysis for primary outcome in patients with diabetes mellitus.

82 paired readings were analysed from 29 individuals with diabetes mellitus. The bias (Difference) in the first 24h after CGM sensor placement was 0.51 mmol/l (95%CI:0.13-0.90). The Lower Limit of Agreement was -2.90 mmol/l (95%CI:-3.56 to -2.20). The Upper Limit of Agreement was 3.93 mmol/l (95%CI:3.27-4.58). BG-blood glucose; CGM-continuous glucose monitor.

# Supplementary Figure 3. Sensitivity analysis for primary outcome in patients who received vasopressor infusions during the first 24h after sensor placement.

285 paired readings were analysed from 95 individuals. The bias (Difference) in the first 24h after CGM sensor placement was 0.36 mmol/l (95%CI:0.20-0.53), with lower LoA = -2.43 mmol/l (95%CI: 2.72 to -2.15) and upper LoA = 3.16 mmol/l (95%CI: 2.88-3.45). BG- blood glucose; CGM- continuous glucose monitor.

# Supplementary Figure 4. Paired comparisons with CGM for 873 blood glucose measurements including and beyond 24h.

**Standard 95.0% LCL 95.0% UCL**

**Variable Count Mean Deviation of Mean of Mean**

──────────────────────────────────────────────────────────────────────────────────────────────────────────────

BG 873 7.82 2.43 7.66 7.98

CGM 873 7.75 2.84 7.56 7.94

Difference 873 0.07 1.43 -0.03 0.16

──────────────────────────────────────────────────────────────────────────────────────────────────────────────

# Supplementary Figure 5. Comprehensive error grid analyses for pre-surgery paired comparisons.


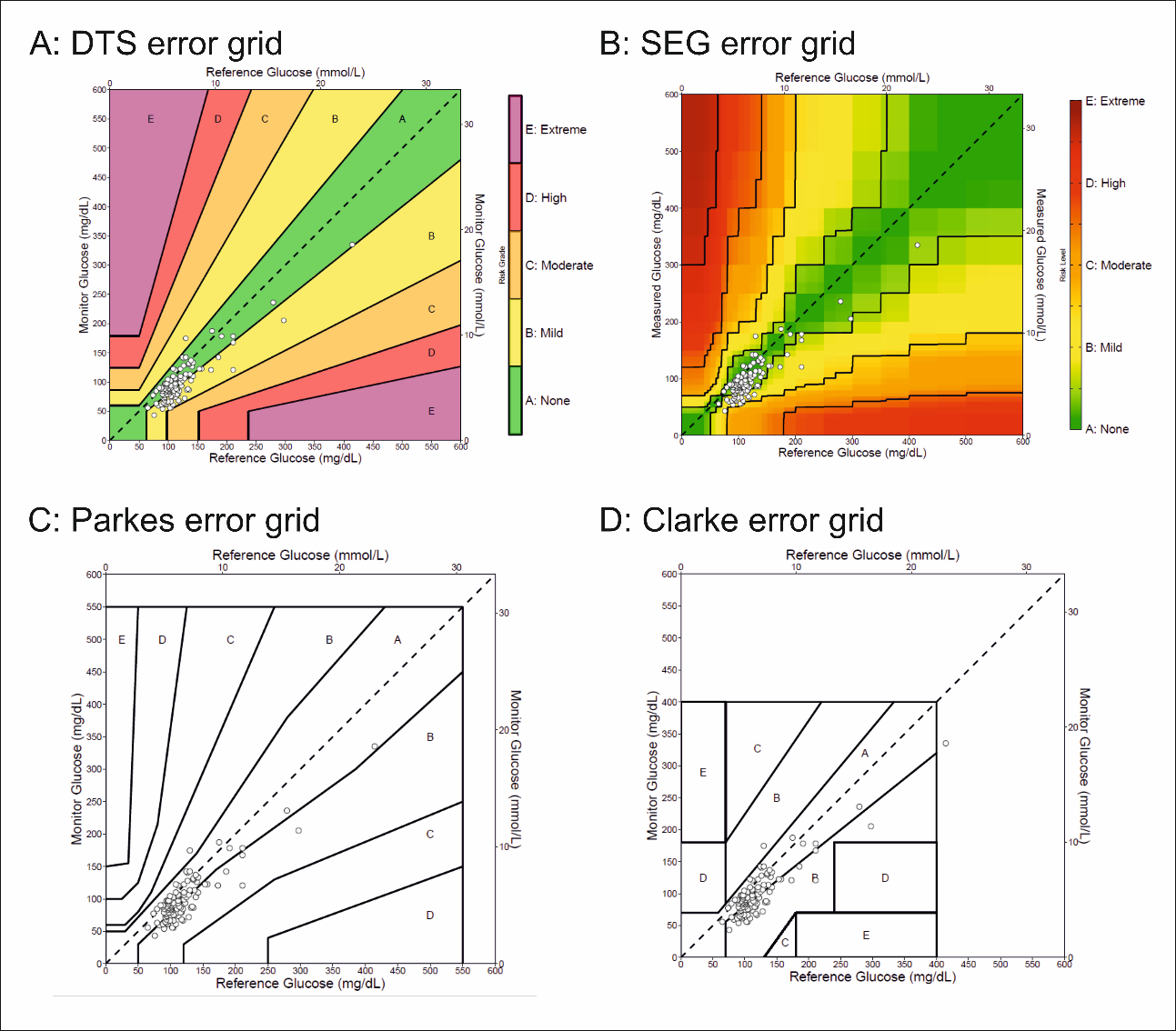


# Supplementary Figure 6. Comprehensive error grid analyses for end of surgery paired comparisons.


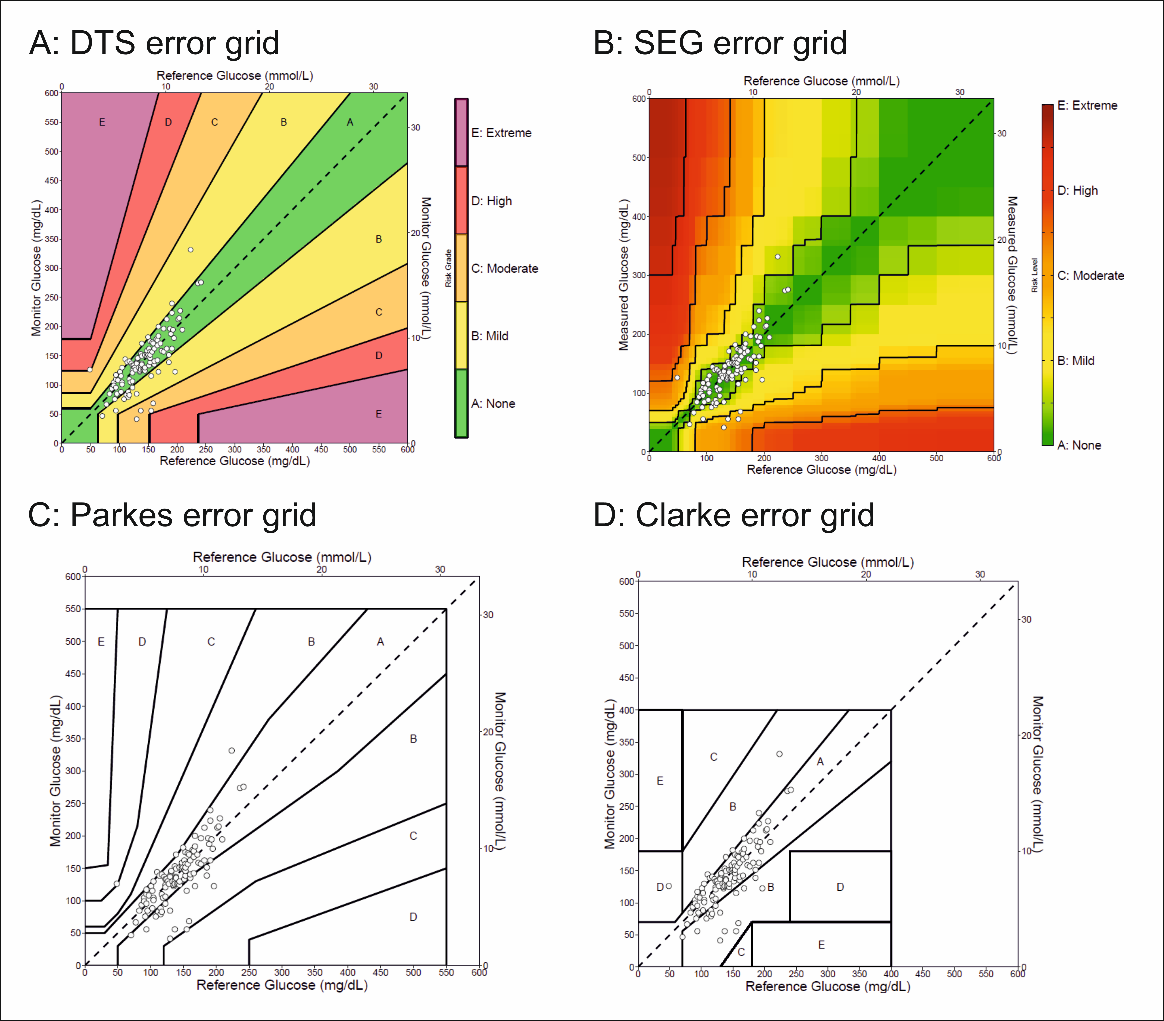


# Supplementary Figure 7. Comprehensive error grid analyses for morning after surgery paired comparisons.


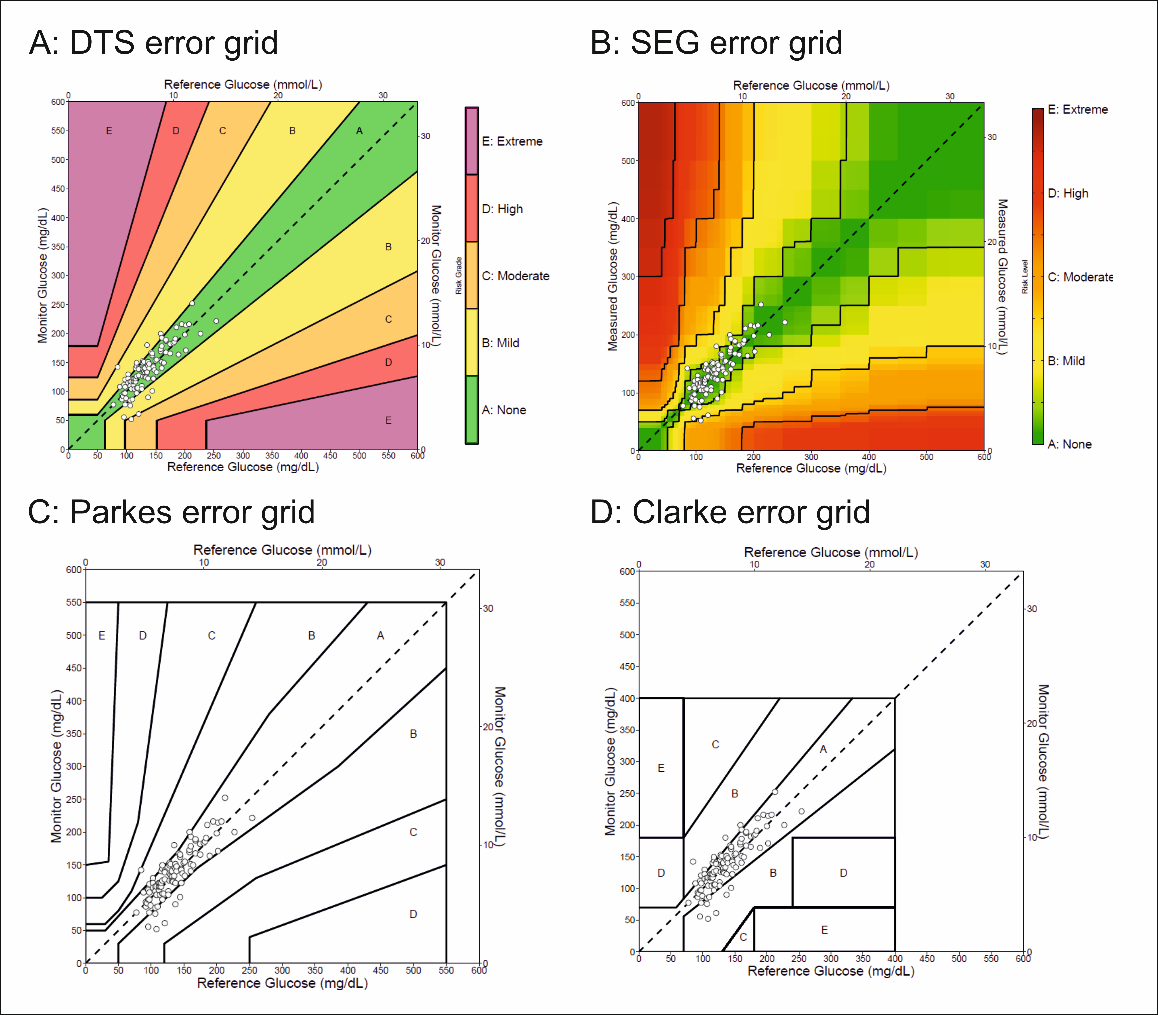


# Supplementary Figure 8. DTS error grid analyses for all paired comparisons throughout surgical period.


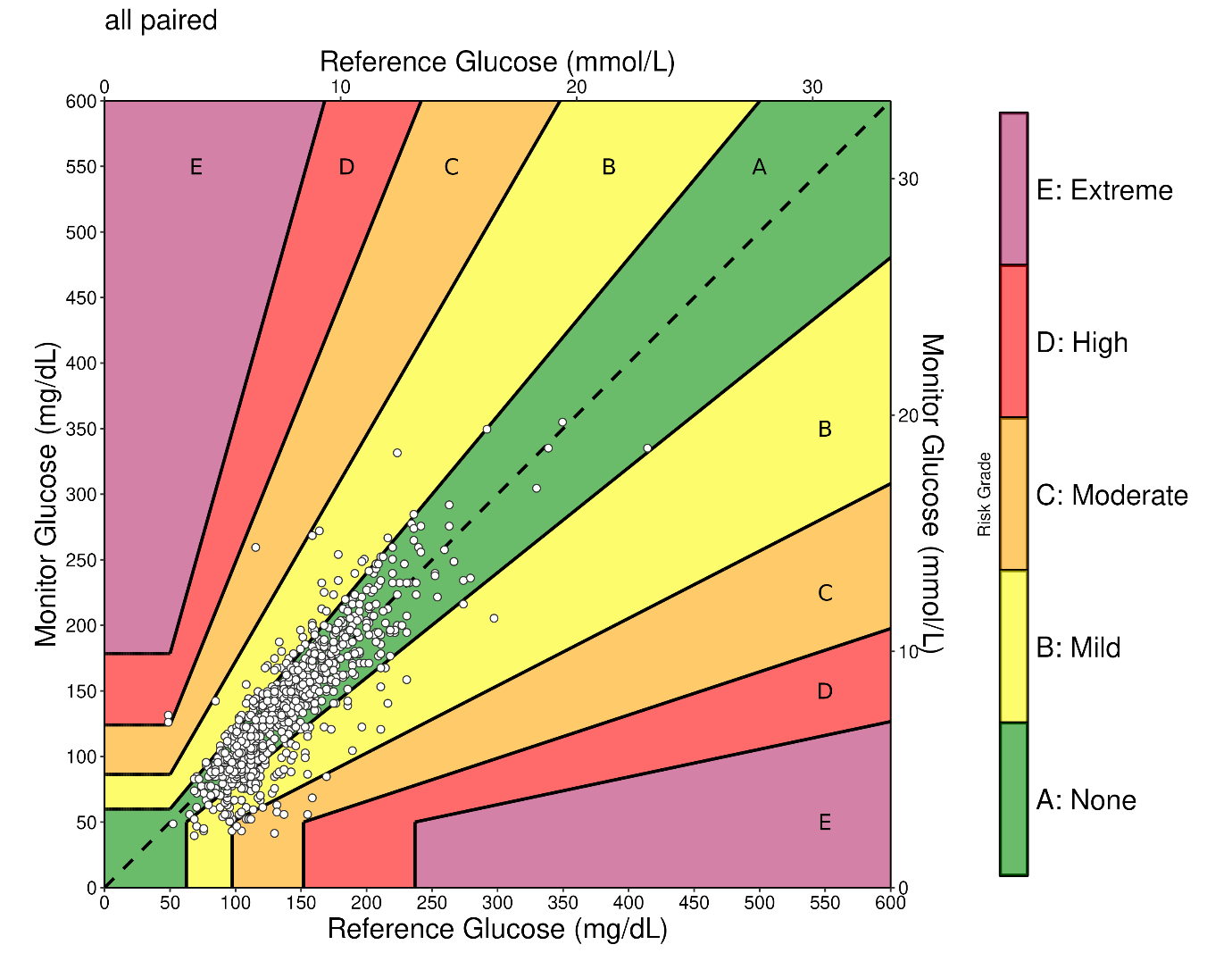


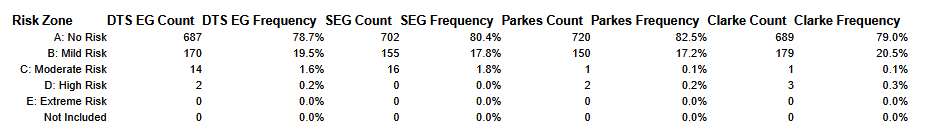

Supplement: Multimedia component 1 [file mmc1.docx]
